# Prognostic model based on m6A-associated lncRNAs in esophageal cancer

**DOI:** 10.3389/fendo.2022.947708

**Published:** 2022-08-30

**Authors:** Weidong Wang, Danhong Dong, Pengfei Yu, Tong Chen, Ruiqi Gao, Jiangpeng Wei, Zhenchang Mo, Haikun Zhou, Qinchuan Yang, Chao Yue, Xisheng Yang, Xiaohua Li, Gang Ji

**Affiliations:** Department of Digestive Surgery, Xijing Hospital, Air Force Military Medical University, Xi’an, China

**Keywords:** esophageal cancer, bioinformatics, lncRNA, m6A, prognostic signature

## Abstract

**Background:**

This research aimed to build an m6A-associated lncRNA prognostic model of esophageal cancer that can be used to predict outcome in esophageal cancer patients.

**Methods:**

RNA sequencing transcriptome data and clinical information about patients with esophageal cancer were obtained according to TCGA. Twenty-four m6A-associated genes were selected based on previous studies. m6A-associated lncRNAs were determined through Pearson correlation analysis. Three m6A-associated lncRNA prognostic signatures were built through analysis of the training set using univariate, LASSO, and multivariate Cox regression. To validate the stabilization of the risk signature, Kaplan–Meier and ROC curve analyses were performed on the testing and complete sets. The prognoses of EC patients were predicted quantitatively by building a nomogram. GSEA was conducted to analyze the underlying signaling pathways and biological processes. To identify the underlying mechanisms through which the lncRNAs act, we constructed a PPI network and a ceRNA network and conducted GO and KEGG pathway analyses. EC samples were evaluated using the ESTIMATE algorithm to compute stromal, immune, and estimate scores. The ssGSEA algorithm was used to quantitatively infer immune cell infiltration and immune functions. The TIDE algorithm was performed to simulate immune evasion and predict the response to immunotherapy.

**Results:**

We identified and validated an m6A-associated lncRNA risk model in EC that could correctly and reliably predict the OS of EC patients. The ceRNA network, PPI network, and GO and KEGG pathway analyses confirmed and the underlying mechanisms and functions provided enlightenment regarding therapeutic strategies for EC. Immunotherapy responses were better in the low-risk subgroup, and PD-1 and CTLA4 checkpoint immunotherapy benefited the patients in the low-risk subgroup.

**Conclusions:**

We constructed a new m6A-related lncRNA prognostic risk model of EC, based on three m6A-related lncRNAs: LINC01612, AC025166.1 and AC016876.2, that can predict the prognoses of EC patients.

## Introduction

Esophageal cancer (EC) ranks eighth and sixth in morbidity and mortality, respectively, among all cancers worldwide (1). Diagnosis and treatment strategies for EC are continually being improved. Treatments for EC include surgery or endoscopic resection, chemotherapy and radiotherapy and immunotherapy (2, 3). However, the prognoses of EC patients are poor, and disease carries a 5-year survival rate of 15% - 25% (4). Hence, the development of a prognostic risk model that can be used to identify high-risk EC patients is urgently needed and may help boost overall survival among EC patients. At the same time, it is vitally necessary to determine novel biological markers that could be effective therapeutic targets for EC patients.

lncRNAs, a set of transcripts with lengths of over 200 nucleotides, lack the potential for protein coding (5, 6). Through transcription and posttranscriptional regulation, lncRNAs participate in various cellular processes, including cell differentiation and proliferation. The anomalous expression of lncRNAs is correlated with the development of neoplasms (7, 8). Among their functions, lncRNAs play vital roles in the carcinogenesis and progression in EC (9). For example, Tan et al. (10) reported that lncRNA H19, as an oncogene, boosts EC cell multiplication and metastasis. Liang et al. (11) indicated that lncRNA CASC9 may boost EC metastasis by upregulating LAMC2 expression levels through its interaction with CREB binding protein.

N6 methyladenosine (m6A) modification plays a vital role in the development of various cancers (12–14). N6 methyladenosine (m6A) RNA modification, which is catalyzed by m6A demethylases (erasers) and m6A methyltransferases (writers), is a dynamic and reversible process. In addition, m6A RNA modification can interact with m6A-binding proteins (readers) to perform a variety of functions (15–17). Previous studies have shown that m6A participates in biological activities associated with the development of EC (18, 19). For instance, METTL3-mediated m6A mRNA modification is significant in the progression of EC through the Notch signaling pathway (20). The m6A eraser ALKBH5 inhibits malignancy in EC by regulating the biogenesis of microRNA and the expression of RAI1 (21). However, the role of m6A-associated lncRNAs in the progression of EC remains to be clarified, and the identification of m6A modification-associated lncRNA biomarkers is significant for the early detection of EC.

In the current study, data on EC patients were acquired from The Cancer Genome Atlas (TCGA) database and subjected to statistical and bioinformatic analyses. We constructed an m6A-associated lncRNA prognostic risk model that is of great diagnostic effectiveness in the evaluation of EC patients. The prognoses of EC patients could be correctly predicted by this signature, which is based on three m6A-associated lncRNAs including LINC01612, AC025166.1 and AC016876.2. Furthermore, the prognoses of patients with EC could be quantitatively predicted by building a nomogram. Ultimately, we built ceRNA and PPI networks and used them to search for novel biomarkers and biological mechanisms through which lncRNAs act.

## Materials and methods

### Data extraction and identification of m6A−associated genes

We collected the expression profiles, including RNA sequencing transcriptomic data in the form of FPKM, and clinical data of EC patients acquired from TCGA (https://cancergenome.nih.gov/). Finally, 162 tumor samples and 11 normal samples were included in the current study. Based on previous researches (16, 22, 23), 24 m6A-related genes were chosen for analysis in the current study. The genes included the following: readers: YTHDF2, YTHDF1, YTHDC2, YTHDF3, YTHDC1, LRPPRC, IGF2BP3, IGF2BP2, IGF2BP1, HNRNPA2B1, FMR1 (FMRP), HNRNPC, and RBMX; erasers: FTO and ALKBH5; and writers: METTL16, METTL14, METTL3, METTL5, ZC3H13, WTAP, RBM15B, KIAA1429 (VIRMA), and RBM15.

### Identification of m6A−associated lncRNAs

We identified 14,056 EC-associated lncRNAs on the basis of the lncRNA annotation file in TCGA. We performed differential analysis of the EC-associated lncRNAs using the “limma” R package (|logFC| > 1, p value< 0.05). According to the results, 1207 of these lncRNAs were identified as significantly different. Pearson correlation analysis of the 24 m6A-associated genes and the annotated lncRNAs was then conducted for lncRNAs for which the |Pearson correlation coefficient| was greater than 0.4 (p< 0.001). Subsequently, 276 m6A-associated lncRNAs were identified for subsequent analyses. Finally, 161 cases in which clinicopathological data and lncRNA expression data were combined were included in this study.

### Bioinformatics analysis

The 161 patients were divided into two sets at random in a ratio of 3:2, yielding a training set consisting of 97 samples and a testing set consisting of 64 samples. The training and testing sets were used to establish and evaluate the prognostic signature, respectively. The significant m6A-associated lncRNAs were determined by univariate Cox regression analysis. The most significant prognostic m6A-associated lncRNAs were chosen through LASSO regression analysis. LASSO regression analysis has the potential to reduce overfitting of the risk model. Multivariate Cox regression analysis was performed to determine the independent prognostic factors. Based on the results of that analysis, a prognostic signature that included three m6A-associated lncRNAs was identified. The risk score (RS) was computed using the following formula: $\text{RS}=\sum_{\text{i}=1}^{\text{n}}\text{coef}(\text{i})*\text{X}(\text{i})$ ; coef(i) refers to the lncRNA coefficient, and X(i) represents the expression level of each lncRNA in this model. The median RS was used as a cut-off value to divide the EC patients into high- and low-risk subgroups. Based on Kaplan-Meier survival curves and receiver operating characteristic curves, we estimated the predictive effectiveness of the model. On the basis of the RS values and clinical parameters, the prognoses of EC patients were predicted by constructing a novel nomogram. The C-index was then calculated, and the calibration curves were plotted to estimate the prediction capacity of the nomogram. We also conducted gene set enrichment analysis (GSEA) for each of the two risk subgroups in the EC TCGA set to determine the possible biological activities and signaling pathway enrichment of the differentially expressed genes. Subsequently, we used the “WGCNA package” in R 4.0.4 to build gene co-expression networks according to the expression profiles of mRNA and lncRNA of EC patients acquired from TCGA portal. We constructed the gene co-expression networks by referring to previously reported methods (24). We used the FPKM method for data normalization and the dynamic tree cut method to set the setting parameters. A ceRNA network based on WGCNA and m6A-associated lncRNAs, was constructed to clarify the mechanisms through which m6A-associated lncRNAs adjust the expression of mRNA through sponging miRNAs in EC. We selected 11 lncRNAs as m6A-related lncRNAs by intersecting the differentially expressed lncRNAs (DElncRNAs) in the EC dataset from TCGA with the lncRNAs from the MEturquoise module. Based on the miRcode database, we predicted target miRNAs that interacted with 11 lncRNAs and identified 57 pairs of interactions between 19 miRNAs and 11 lncRNAs. Three different databases, including miRDB (25), miRTarBase (26), and TargetScan (27), were utilized to predict the relationships between these target mRNAs and 19 miRNAs and to determine the target mRNAs of these miRNAs. The ceRNA network composed of the twelve m6A-associated lncRNAs, the targeted miRNAs and mRNAs was plotted *via* Cytoscape software (28). Through the STRING database, we established a PPI network on the basis of these 90 targeted mRNAs (29). Then, Gene Ontology (GO) and Kyoto Encyclopedia of Genes and Genomes (KEGG) analyses were then conducted to identify the underlying biological processes associated with these genes.

Stromal, estimate, and immune scores of the EC samples were computed using the ESTIMATE algorithm in the R package. A single-sample gene set enrichment analysis (ssGSEA) algorithm was implemented to quantitatively infer immune cell infiltration and immune functions. In addition, boxplots were drawn for analysis of the expression status of common immune checkpoints in the two subgroups. Immune evasion was simulated, and the response of patients to immunotherapy was predicted accurately using the tumor immune dysfunction and exclusion (TIDE) algorithm (30). No-responders were defined as patients whose TIDE scores were more than 0.2, and responders were defined as patients whose TIDE scores were less than 0.2. A subclass mapping algorithm was used to evaluate the similarities in response to immunotherapies among the patients who responded to immunotherapies and between the two groups of EC patients (31).

### Statistical analysis

R 4.0.4 and Perl language 5.30.2 were used in statistical analyses and data handling. The K-M curve and the log-rank test were used to evaluate OS. Univariate, LASSO and multivariate Cox regression analyses were also conducted to determine and validate the prognostic significance of m6A-associated lncRNAs. The ROC curve and the area under the ROC curve (AUC) were used to estimate the prognostic performance of the lncRNA model, including its sensitivity and reliability. P< 0.05 was considered statistically as significant in statistics, and 95% was set as the value of the confidence interval (CI) value.

## Results

### Establishment of a prognostic signature based on 3 m6A-associated lncRNAs in the training set

**Figure 1:**
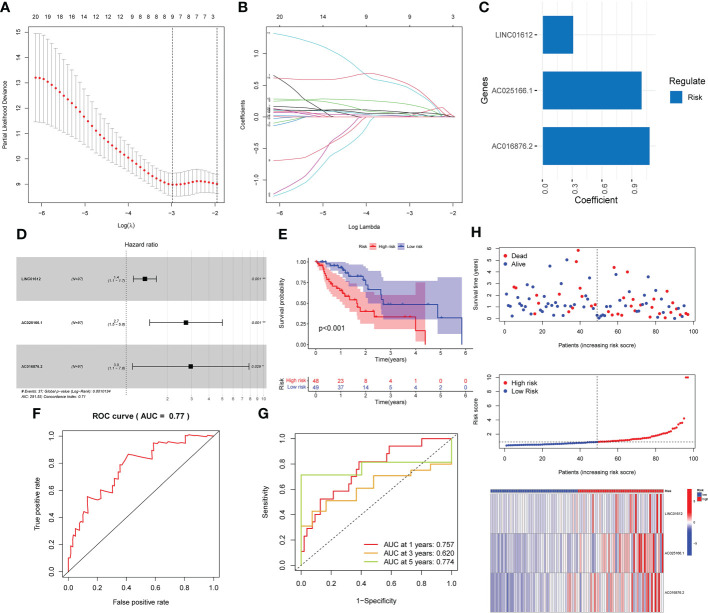
Development of a prognostic risk model based on the expression of three lncRNAs associated with m6A in the training set. **(A–C)** Three lncRNAs associated with m6A were identified by LASSO Cox regression analysis. **(D)** Forest plot of three m6A-related lncRNAs. **(E)** K-M survival curves uncovered that EC patients in the low-risk subgroup in the training set possessed a better OS. **(F)** Prognostic signatures in the training set were analyzed using ROC curves to determine their predictive capacity. **(G)** The prediction efficiency of the prognostic risk model for 1 year, 3 years, and 5 years was determined through ROC curve analysis. **(H)** Survival status scatter plot. The distribution of risk scores and the expression profile heatmap of three m6A-associated lncRNAs in the training set are shown. *p< 0.05; **p< 0.01; ***p< 0.001.

A prognostic signature was established through univariate Cox regression analysis of the data in the training set of the EC patients on the basis of the expression levels of the 276 m6A-associated lncRNAs. There was a significant correlation between the expression of 20 m6A-related lncRNAs and the OS of EC patients. Subsequently, we implemented LASSO Cox analysis of the 20 m6A-associated lncRNAs and we determined 9 m6A-associated lncRNAs (**Figures 1A, B**). Three m6A-associated lncRNAs were determined to be independent predictors of survival in EC. The coefficient of each lncRNA was computed using multivariate Cox regression analysis (**Figure 1C**). **Table 2** shows the coefficient of each lncRNA in the prognostic signature. Additionally, LINC01612, AC016876.2 and AC025166.1, hazard ratio (HR) >1, were considered risk factors (**Figure 1D**). We divided the EC patients in the training set into high- and low-risk subgroups on the basis of the median RS as the cut-off value (**Table 1**). K-M survival curves demonstrated that compared to EC patients in the low-risk subgroup, those in the high-risk subgroup possessed worse OS (**Figure 1E**). ROC curve analysis was carried out and displayed that the RS possessed a stable predictive capacity (AUC value = 0.77; **Figure 1F**). Additionally, the AUC values of the time-dependent 1-, 3-, and 5- year ROC curves in **Figure 1G** were 0.757, 0.620 and 0.774, respectively. Moreover, the survival status, RS distributions and 3 m6A-associated lncRNA expression profiles in the training set are plotted in **Figure 1H**.

**Table 1:** Clinical characteristics of the training and testing sets of EC patients.

Characteristic	n (%)			P-value
	Complete set	Training set	Testing set	
age				
≤65	96 (61.49%)	57 (57.58%)	42 (62.62%)	0.381
>65	62 (38.51%)	40 (42.42%)	22 (34.38%)	
sex				
male	138 (85.71%)	82 (84.54%)	56 (87.50%)	0.599
female	23 (14.29%)	15 (15.46%)	8 (12.50%)	
grade				
G1-2	82 (50.93%)	47 (48.45%)	35 (54.69%)	0.347
G3	44 (27.33%)	29 (29.90%)	15 (23.44%)	
GX	35 (21.74%)	21 (21.65%)	14 (21.87%)	
stage				
Stage I-II	85 (52.80%)	57 (58.76%)	28 (43.75%)	0.084
Stage III-IV	57 (35.40%)	30 (37.04%)	27 (42.19%)	
unknow	19 (11.80%)	10 (6.17%)	9 (14.06%)	
T				
T1-2	65 (40.37%)	41 (42.27%)	24 (37.5%)	0.638
T3-4	81 (50.31%)	48 (49.48%)	33 (51.56%)	
unknow	15 (9.32%)	8 (8.25%)	7 (10.94%)	
N				
N0	66 (40.99%)	42 (43.30%)	24 (37.50%)	0.567
N1-3	78 (48.45%)	46 (47.42%)	32 (50.00%)	
NX	2 (1.24%)	1 (1.03%)	1 (1.56%)	
unknow	15 (9.32%)	8 (8.25%)	7 (10.94%)	
M				
M0	121 (75.15%)	75 (77.32%)	46 (71.87%)	0.977
M1	8 (4.97%)	5 (5.15%)	3 (4.69%)	
MX	14 (8.70%)	8 (8.25%)	6 (9.38%)	
unknow	18 (11.18%)	9 (9.28%)	9 (14.06%)	

**Table 2:** The coefficient of each lncRNA in the prognostic signature.

Gene	Coef
LINC01612	0.315708501
AC025166.1	1.003581598
AC016876.2	1.083812387

**Figure 2:**
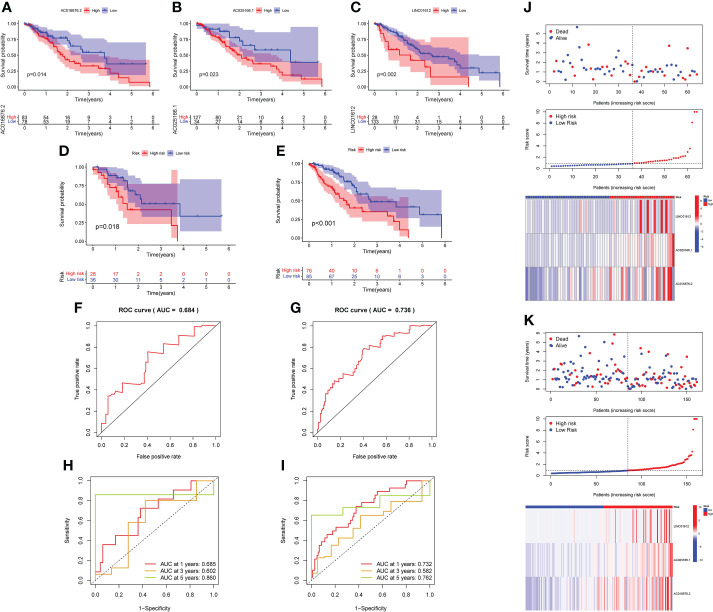
K-M survival analysis was conducted to assess the roles of the 3 lncRNAs in prognoses. **(A–C)** K-M survival curves demonstrated that a high level of LINC01612, AC025166.1 and AC016876.2 expression was associated with worse OS in the training set (p< 0.05), validation of the prognostic risk model according to 3 m6A-associated lncRNAs in the testing and complete sets. **(D, E)** K-M survival curves indicated that a subgroup of EC patients at low risk possessed a better OS in the testing and complete sets. **(F, G)** The prognostic risk model was able to predict the outcome using ROC curves in the testing set and complete set. **(H, I)** An analysis of ROC curves was carried out to determine the prognostic risk model’s predictive effectiveness for survival rates at 1, 3, and 5 years in the testing and complete sets. **(J, K)** Survival status scatter plot, risk score distribution and heatmap of the expression profiles three m6A-associated lncRNAs in the testing and complete sets.

K-M survival analysis was implemented to assess the roles of the 3 lncRNAs in patient prognoses. The results demonstrated that there was a significant correlation between the high expression of LINC01612, AC025166.1 and AC016876.2 and worse OS in the training set (p value< 0.05; **Figures 2A–C**).

### Validation of the prognostic risk model consisting of 3 m6A-associated lncRNAs in the testing set

Based on the median RS as the cut-off value, the EC patients in testing and complete sets were split into high- and low-risk subgroups for verification of the predictive capacity of the model. K-M survival analysis demonstrated that EC patients in the low-risk subgroup possessed better OS in both the testing and complete sets than those in the high-risk subgroup (**Figures 2D, E**). The ROC curves displayed that the RS possessed a dependable and steady predictive ability in both the testing set (AUC value = 0.684; **Figure 2F**) and the complete set (AUC = 0.736; **Figure 2G**). Moreover, the AUC values of the time-dependent 1-, 3-, and 5 year- ROC curves were 0.685, 0.602 and 0.860 for the testing set in **Figure 2H**, respectively. As shown in **Figure 2I**, the time-dependent ROC curve revealed that the AUC values of the 1-, 3-, and 5- year ROC curves for the prognostic signature in the complete set were 0.732, 0.582 and 0.762, respectively. The survival status, RS distributions and expression profiles of 3 m6A-related lncRNAs in the testing and complete sets are plotted in **Figures 2J, K**. The results of these plots for the testing set and complete set were consistent with those obtained for the training set, showing that the prognostic risk model possessed a stable and reliable predictive capacity.

### Assessment of the clinical value of the prognostic model according to 3 m6A-associated lncRNAs in EC patients

**Figure 3:**
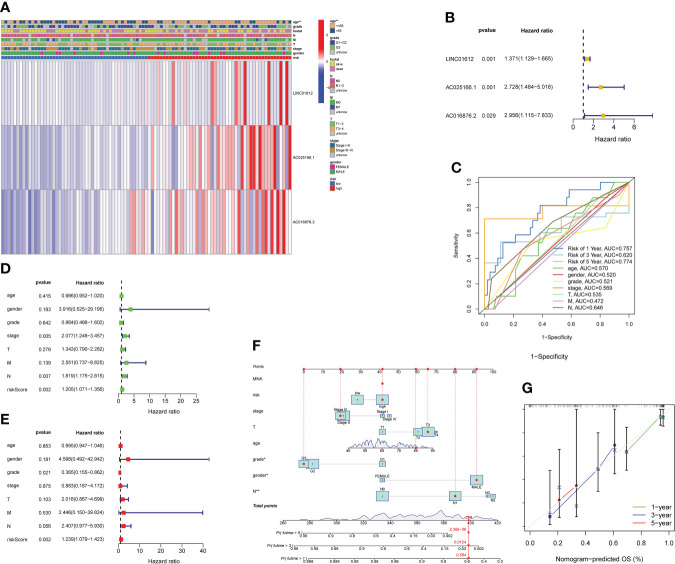
Evaluation of the value of prognostic risk model according to 3 m6A-related lncRNAs in EC patients in clinical practice. **(A)** Heatmap revealing the correlations between the levels of expression of 3 m6A-associated prognostic lncRNAs and clinicopathological characteristics, including age, sex, grade, TNM stage and AJCC stage, in the two subgroups. Significant differences in age were found between the two subgroups (p< 0.01). **(B)** As shown in the forest plot, we found that LINC01612, AC025166.1 and AC016876.2, HR values > 1, were risk factors in EC patients. **(C)** Multivariate ROC curves revealed that values of AUC for prognostic risk model of 1- and 5- year ROC curves were 0.757 and 0.774, reflecting their superior performance compared to conventional clinical features. **(D)** According to the results of a univariate Cox regression analysis, stage, RS, and N stage were all associated with prognosis. **(E)** Multivariate Cox regression analysis revealed that grade and RS were independent predictors of survival. **(F)** Nomogram, including both the prognostic signature and clinicopathological features, was designed to quantitatively estimate the prognoses of EC patients. **(G)** Nomogram calibration curve showing the coherence between predicted and observed OS for 1-, 3-, and 5-year periods. **p< 0.01.

We evaluated the predictive value of the signature comprising 3 m6A-related lncRNAs in EC patients stratified by the clinicopathological characteristics, consisting of age, sex, grade, TNM stage and AJCC stage. The heatmap revealed the correlations between the levels of expression of 3 m6A-associated lncRNAs and clinical features in the two subgroups (**Figure 3A**). Significant differences in age were found between the high- and low-risk subgroups (p value< 0.01). As shown in the forest plot, we found that LINC01612, AC025166.1 and AC016876.2, with HR values > 1, were risk factors in EC patients (**Figure 3B**). Therefore, the three m6A-associated lncRNAs included in the prognostic model have a dependable and stable prognostic capacity. Subsequently, we obtained a multivariate ROC curve of the RS values from the prognostic signature and conventional clinical features, as shown in **Figure 3C**. The results indicated that the AUC values of the prognostic signature consisting of 3 m6A-associated lncRNAs of the 1-, 3-, and 5- year ROC curves were 0.757, 0.620 and 0.774, respectively. Especially the AUC values of the prognostic signature of 1- and 5- year ROC curves reflect their superior performance compared to conventional clinical features, including age (AUC=0.570), sex (AUC=0.520), grade (AUC=0.521), AJCC stage (AUC=0.569), T stage (AUC=0.535), M stage (AUC=0.472), and N stage (AUC=0.646). Additionally, univariate and multivariate Cox regression analyses of the prognostic signature comprising m6A-associated lncRNAs and clinical features, such as age, sex, TNM stage and AJCC stage, were conducted to estimate the clinical application value of the developed prognostic signature. As shown in **Figure 3D**, univariate Cox regression analysis indicated that RS, stage, and N stage were prognosis-associated variables. As shown in **Figure 3E**, multivariate Cox regression analysis indicated that the RS and grade were independent predictors. These results revealed that the prognostic signature consisting of 3 m6A-associated lncRNAs could independently predict the survival outcomes of EC patients (p < 0.01). Ultimately, a nomogram including both the prognostic signature and clinicopathological features was built to quantitatively predict the prognoses of EC patients (**Figure 3F**). The C-index value of this nomogram was 0.696. The calibration curve of the nomogram displayed coherence between the prediction and observation of 1-, 3-, and 5-year OS and confirmed the accuracy and reliability of the prognostic nomogram (**Figure 3G**).

### Stratification analysis according to the patients’ clinicopathological characteristics

**Figure 4:**
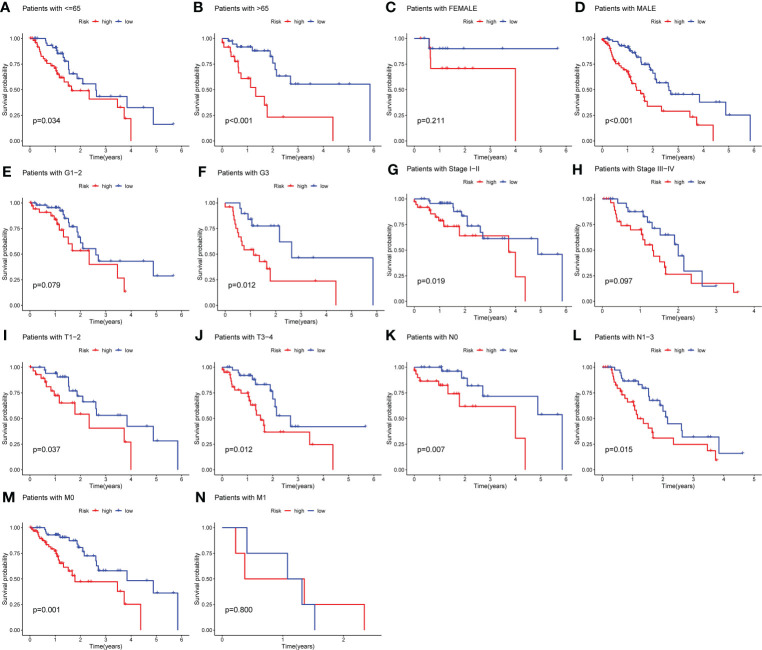
Stratification analysis according to different clinicopathological characteristics. **(A–N)** Stratification survival analysis was implemented on the basis of clinicopathological characteristics, including age (age ≤ 65 vs. age > 65), sex (male vs. female), grade (G1–G2 vs. G3), stage (stages I–II vs. III–IV), stage T (T1–T2 vs. T3–T4), stage M (M0 vs. M1), and stage N (N0 vs. N1). K-M survival curves uncovered that for patients with the characteristics of age > 65, age ≤ 65, male sex, grade 3, stage I–II, T1-2, T3-4, N0, N1-3, and M0, there was a better OS among EC patients in the low-risk subgroup compared to those in the high-risk subgroup (p< 0.05).

To verify the predictive capacity of the m6A-associated lncRNA prognostic risk model as a prognostic factor of OS in high- and low-risk patient subgroups, stratification survival analysis was conducted based on clinicopathological characteristics, including age (age ≤ 65 vs. age > 65), sex (male vs. female), grade (G1–G2 vs. G3), stage (stages I–II vs. III–IV), stage T (T1–T2 vs. T3–T4), stage N (N0 vs. N1), and stage M (M0 vs. M1). K-M survival curves revealed that for patients with the characteristics of age ≤ 65, age > 65, male sex, grade 3, stage I–II, T1-2, T3-4, N0, N1-3, and M0, those in the low-risk subgroup possessed better OS than those in the high-risk subgroup (p< 0.05; **Figures 4A–N**).

### GSEA between the high-risk and low-risk subgroups

**Figure 5:**
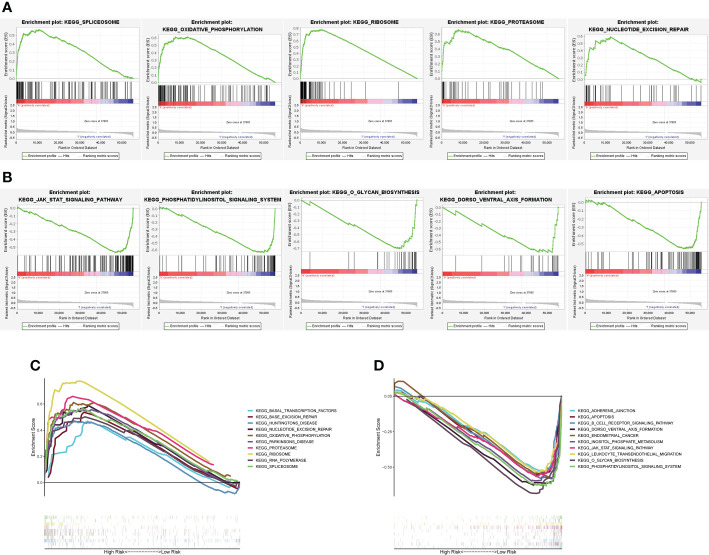
GSEA between the high- and low-risk subgroups. **(A)** GSEA uncovered that among the high-risk subgroup, altered genes were primarily enriched in spliceosome genes, oxidative-phosphorylation, ribosome, proteasome and nucleotide-excision-repair. **(B)** An analysis of GSEA revealed that altered genes in the low-risk group were primarily enriched in Jak-stat-signaling-pathway, phosphatidylinositol-signaling-system, o-glycan-biosynthesis, dorso-ventral-axis-formation, and apoptosis. **(C, D)** First 10 signaling pathways by KEGG analysis in the two subgroups.

To determine the possible signaling pathways and cellular processes in relation to molecular heterogeneity, we performed GSEA between the two subgroups. The results showed that EC in the high-risk subgroup exhibited enrichment of pathways involved in the spliceosome, oxidative-phosphorylation, ribosome, proteasome and nucleotide-excision-repair (**Figure 5A**). Additionally, EC in the low-risk subgroup exhibited the enrichment of pathways involved in jak-stat-signaling-pathway, phosphatidylinositol-signaling-system, o-glycan-biosynthesis, dorso-ventral-axis-formation, and apoptosis (**Figure 5B**). We also determined the top 10 enriched signaling pathways acquired in KEGG analysis in the high- and low-risk subgroup (**Figures 5C, D**). These results provide a novel basis for developing individualized treatments for EC patients in different risk subgroups.

### CeRNA network construction, PPI network analysis and functional enrichment analysis

**Figure 6:**
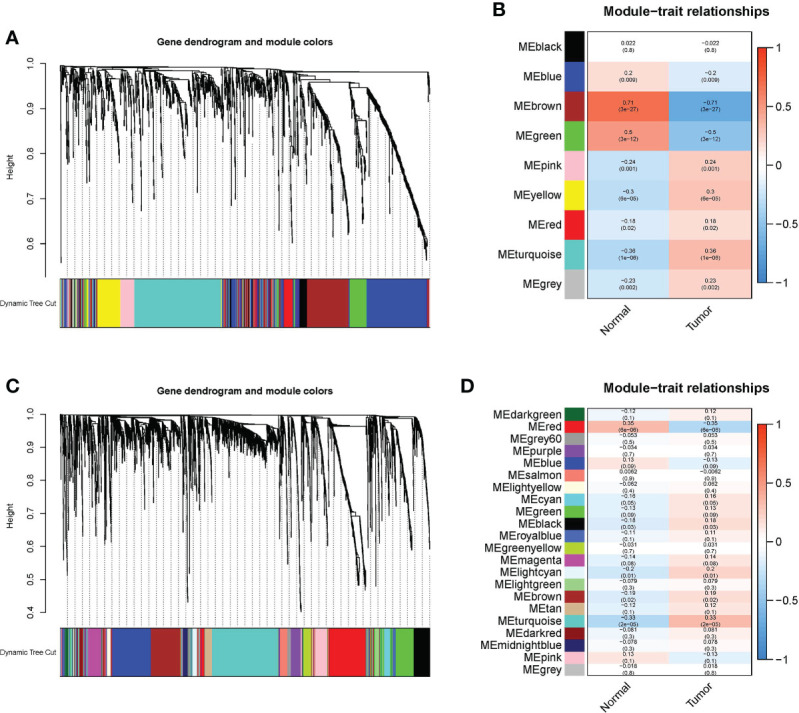
WGCNA was conducted to determine modules related to the clinical characteristics of EC. **(A)** In the EC module, hierarchical clustering dendrograms of determined lncRNAs are shown. **(B)** Correlations between Eigengenes of lncRNAs and clinical characteristics of EC in a heatmap. In each colored module, the association and p value are listed, and the MEbrown module with the highest correlation coefficient is selected. **(C)** In the EC module, hierarchical clustering dendrograms of determined mRNAs are shown. **(D)** Correlations between eigengenes of mRNAs and clinical characteristics of EC in a heatmap. In each colored module, the association and p value are listed, and the MEred module with the highest correlation coefficient is selected.

A ceRNA network, based on WGCNA and m6A-related lncRNAs, was constructed to clarify the mechanism through which m6A-associated lncRNAs regulate the expression of mRNAs through miRNAs in EC. Additionally, a PPI network was constructed *via* the STRING database. As shown in **Figures 6A, B**, identification of lncRNAs in modules related to the clinical characteristics of EC was done using WGCNA, and the MEturquoise module that possessed the maximum correlation coefficient was chosen. Subsequently, we selected the mRNAs in the MEgreen module in the same way (**Figures 6C, D**).

**Figure 7:**
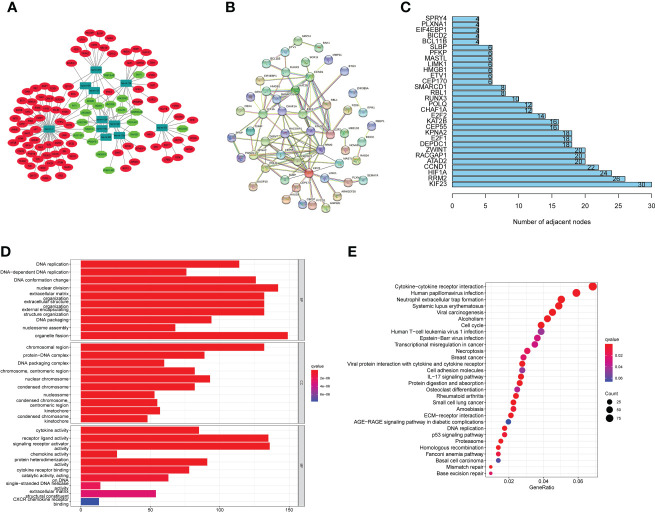
CeRNA network construction, PPI network analysis and functional enrichment analysis. **(A)** CeRNA network according to the expression profiles of 19 miRNAs, 11 lncRNAs, and 90 mRNAs in EC patients. **(B)** PPI analysis was performed on these 90 target mRNAs. **(C)** Bar chart displaying the a PPI network with connection nodes for the top 30 targeted mRNAs. **(D)** An analysis of KEGG pathways shows that targeted mRNAs enriched signaling pathways. **(E)** GO enrichment analysis of targeted mRNAs uncovered the BP, CC, and MF.

We built a ceRNA network based on the expression profiles of 19 miRNAs, 11 lncRNAs, and 90 mRNAs in EC patients (**Figure 7A**). PPI analysis was then performed on these 90 target mRNAs (**Figure 7B**). The bar chart displays the connection nodes of the first 30 targeted mRNAs in the PPI network (**Figure 7C**). To determine the underlying functions of m6A-associated lncRNAs in EC, GO and KEGG analyses were performed on the 90 targeted mRNAs. KEGG pathway analysis revealed that the signaling pathways enriched in the targeted mRNAs included systemic lupus erythematosus, cell cycle, DNA replication, neutrophil extracellular trap formation, and cytokine-cytokine receptor interaction (**Figure 7D**). GO enrichment analysis of targeted mRNAs indicated that the top three biological processes (BPs) included DNA replication, DNA-dependent DNA replication and DNA conformation change. The top three GO terms for cell components (CC) were chromosomal region, protein-DNA complex and DNA packaging complex. The top three GO terms for molecular functions (MF) were cytokine activity, receptor ligand activity and signaling receptor activator activity (**Figure 7E**).

### Relationship between immune infiltration and the prognostic risk model of m6A-associated lncRNAs in EC patients

**Figure 8:**
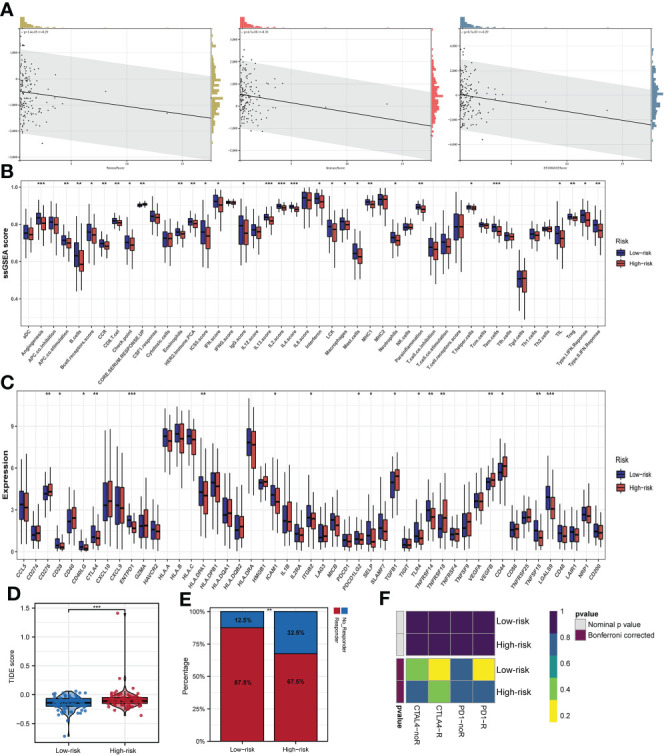
Immune infiltration and prognostic significance of m6A-associated lncRNAs in EC patients. **(A)** Risk score value is related to stromal score, immune score, and estimate score. **(B)** Infiltrations of immune cells and immune function quantified by the ssGSEA Z-score among patients with EC. **(C)** The expression status of common immune checkpoints between subgroups with high- and low-risks. **(D)** TIDE score between the two subgroups. **(E)** The distribution of immunotherapeutic responses between the two subgroups. **(F)** Subclass mapping algorithm between immunotherapy-responding patients and EC patients in two subgroups *p<0.05; **p<0.01; ***p<0.001.

Comparison of the stromal score, estimate score, and immune score of the high- and low-risk subgroups, indicated that the patients in the high-risk subgroup possessed lower status of the above three scores (**Figure 8A**). As shown in **Figure 8B**, there were significant differences in the distribution of ssGSEA Z-score of 29 of the 42 immune signatures between the two subgroups such as angiogenesis, APC co-stimulation, B cells and so on. Evidence is mounting that recruiting B cells and obtaining inhibitory activity in tumor bed may be an important way for B cells to play an anti-tumor immune role (32). Most notably, our findings suggested that the high expression of B cells in low-risk subgroup may partially explain the better prognosis of low-risk subgroup compared to high-risk subgroup. Subsequently, 49 common immune checkpoints were selected to evaluate their correlation with the prognostic signature of m6A-associated lncRNAs. The boxplots displayed that the expression status of 18 immune-checkpoints (CD276, CD28, CD40LG, CDLA4, ENTPD1, HLA-DPA1, ICAM1, ITGB2, PDCD1LG2, SELP, TGFB1, TLR4, TNFRSF14, TNFRSF18, VEGFB, CD44, TNFRSF15 and LGALS9) were significantly different between the two subgroups, which suggested that there may be differences in immunotherapy responses between the two subgroups (**Figure 8C**). Additionally, TIDE analysis uncovered that the EC patients in the low-risk subgroup possessed lower TIDE score than that in the high-risk subgroup, implying that the patients in the low-risk subgroup may have a better respond to immunotherapy (**Figure 8D**). As shown in **Figure 8E**, 87.5% of the patients in the low-risk subgroup responded to immunotherapy. Subclass mapping analysis revealed that PD-1 and CTLA4 checkpoint immunotherapy may benefit patients in the low-risk subgroup (**Figure 8F**).

## Discussion

Esophageal cancer, which possesses a poor prognosis, ranks eighth in incidence and sixth in mortality among all types of tumors worldwide (1, 4). Therefore, it is necessary to identify novel biomarkers that could be effective therapeutic targets in EC patients. Previous studies (33, 34) reported that a signature on the basis of m6A-associated gene expressions had good prediction ability for EC patients. However, there is still no m6A-associated lncRNA prognostic risk model that predicts the prognoses of EC patients. This is the first study to discuss the establishment of a risk model based on the expression of m6A-associated lncRNAs in EC patients.

In this research, we developed a prognostic signature based on the expressions of three m6A-related lncRNAs: LINC01612, AC025166.1 and AC016876.2. To establish a reliable prognostic signature, a total of 161 patients were randomly classified into training and testing sets in a 3:2 ratio. We conducted univariate Cox regression analysis on the training set of EC patients to develop a prognostic signature and uncovered that there was a significant relationship between m6A-associated lncRNA expression and prognosis among EC patients. Additionally, we conducted LASSO and multivariate Cox regression analyses and identified a total of three m6A-assciated lncRNAs as independent predictors of OS in EC. Taking the median RS as the cut-off value, the training set of EC patients was divided into two subgroups: high-risk and low-risk subgroups, and the signature’s predictive effectiveness was assessed using K-M survival curves and ROC curves. The results revealed that EC patients in the low-risk subgroup exhibited better OS than patients in the high-risk subgroup in the training, testing and complete sets. Subsequently, to predict the prognoses of EC patients, we developed a novel nomogram. Ultimately, we explored the underlying biological mechanisms through which these lncRNAs exert their effects by establishing a PPI network and a ceRNA network. In EC, we used GO and KEGG analyses to examine the underlying functions of lncRNAs associated with m6A. Our findings uncovered that the prognostic risk model had a dependable and robust predictive capacity. Additionally, there were significant differences in the distribution of ssGSEA Z-score of 29 immune signatures such as angiogenesis, APC co-stimulation, B cells between the two subgroups. There were significant differences in 18 immune-checkpoints such as CD276, CD28, CD40LG, and so on, were between the two subgroups. Most notably, our results indicated that immunotherapy may be more effective in patients in the low-risk group, and that such patients may even benefit from PD-1 and CTLA4 checkpoint immunotherapy.

The clinical and application value of this prognostic risk model comprising three m6A-associated lncRNAs and clinicopathological features was evaluated through univariate and multivariate Cox regression analyses. The results showed that the prognostic signature based on the expressions of 3 m6A-associated lncRNAs was an independent predictor for the survival outcomes of EC patients. Stratification analysis uncovered that EC patients in the low-risk subgroup possessed better OS than those in the high-risk subgroup. The above findings uncovered that the prognostic model possesses a dependable and steady predictive capacity.

Based on our analysis, three m6A-related lncRNAs, LINC01612, AC025166.1 and AC016876.2, were included in the prognostic signature. K-M survival analysis uncovered that there was a significant correlation between the high expression of LINC01612, AC025166.1 and AC016876.2 and worse OS in EC patients. These results illustrated that LINC01612, AC025166.1 and AC016876.2 may be the promoting factors of EC. These findings suggested that these three m6A-related lncRNAs could be applied as underlying biological markers for the future molecular diagnosis and targeted treatment of EC. However, few previous researches have concerned the roles of the three m6A-associated lncRNAs in the carcinogenesis and progression of EC. Therefore, we hope that our findings will provide enlightenment for future EC research.

However, this study also has several limitations. First, we identified and validated the prognostic signature according to the TCGA database only since no other suitable public database was available. Second, *in vivo* and *in vitro* experiments are also necessary to determine the mechanisms and signaling pathways of m6A-associated lncRNAs in EC in future studies.

In conclusion, we constructed a new m6A-associated lncRNA prognostic risk model of EC, that could predict the prognoses of EC patients as an independent predictor. Additionally, PPI network and ceRNA network were built to determine the underlying biological mechanisms of these lncRNAs. The results of GO and KEGG analyses could also provide enlightenment to confirm the functions of m6A-associated lncRNAs in EC.

## Data availability statement

The datasets presented in this study can be found in online repositories. The names of the repository/repositories and accession number(s) can be found in the article.

## Author contributions

Conceptualization, WW and XL. Methodology, WW, DD, PY, TC.

Software, WW, DD, PY, TC. Validation, WW, DD, PY, TC. Formal analysis, WW, DD, PY, TC. Investigation, RG, ZM, JW, HZ, QY, CY, XY. Resources, RG, ZM, JW, HZ. Data curation, QY, CY, XY. Writing—original draft preparation, WW. Writing—review and editing, WW, XL, and GJ. Visualization, RG, ZM, JW. Supervision, HZ. Project administration, GJ. Funding acquisition, XL, and GJ. All authors contributed to the article and approved the submitted version.

## Funding

This work was funded by grants from the National Natural Science Foundation of China (Young Program 82100680) and the Shaanxi Innovation Team(2021-TD-43).

## Acknowledgments

We thank the TCGA database for the availability of the data.

## Conflict of interest

The authors declare that the research was conducted in the absence of any commercial or financial relationships that could be construed as a potential conflict of interest.

## Publisher’s note

All claims expressed in this article are solely those of the authors and do not necessarily represent those of their affiliated organizations, or those of the publisher, the editors and the reviewers. Any product that may be evaluated in this article, or claim that may be made by its manufacturer, is not guaranteed or endorsed by the publisher.

## Abbreviations

EC, Esophageal cancer; m6A, N6-methyladenosine; lncRNA, long noncoding RNA; OS, the overall survival; TCGA, The Cancer Genome Atlas; RS, risk score; ROC, receiver operating characteristic; AUC, area under the curve; CI, confidence interval; C-index, the concordance index; GSEA, gene set enrichment analysis; KEGG, kyoto encyclopedia of genes and genomes; GO, gene ontology; HR, hazard radio; MF, molecular functions; CC, cell components; BP, biological processes
